# Reduced hypopharyngeal muscle strength in patients with dysphagia symptoms after anterior cervical discectomy and fusion

**DOI:** 10.1186/s13741-026-00691-4

**Published:** 2026-05-07

**Authors:** Chih-Jun Lai, Fon-Yih Tsuang, Jing-Rong Jhuang, Ming-Yen Hsiao, Ya-Jung Cheng, Yeun-Chung Chang, Jo-Yu Chen

**Affiliations:** 1 Department of Anesthesiology, National Taiwan University Hospital, Taipei, Taiwan; 2 Department of Surgery, National Taiwan University Hospital, Taipei, Taiwan; 3 Institute of Statistical Science, Academia Sinica, Taipei, Taiwan; 4 Department of Physical Medicine and Rehabilitation, College of Medicine, National Taiwan University, Taipei, Taiwan; 5 Department of Anesthesiology, College of Medicine, National Taiwan University, Taipei, Taiwan; 6 Department of Medical Imaging, National Taiwan University Hospital, Taipei, Taiwan; 7 Graduate Institute of Clinical Medicine, College of Medicine, National Taiwan University, Taipei, Taiwan

**Keywords:** Dysphagia symptoms, The Eating Assessment Tool-10, Anterior cervical discectomy and fusion, High-resolution impedance manometry, Videofluoroscopy

## Abstract

**Background:**

Dysphagia symptoms are a common complication following anterior cervical discectomy and fusion (ACDF). The Eating Assessment Tool-10 (EAT-10) is widely used to screen for dysphagia symptoms, particularly in patients with scores ≥ 3. However, the underlying pathophysiological mechanisms and specific swallowing muscle impairments associated with dysphagia symptoms after ACDF remain incompletely understood. Videofluoroscopy is considered the gold standard for evaluating swallowing function. High-resolution impedance manometry (HRIM) enables quantitative assessment of pressure dynamics during swallowing. Therefore, this study aimed to identify alterations in pharyngeal contractile function and pathophysiological changes associated with dysphagia symptoms following ACDF using HRIM and videofluoroscopy.

**Methods:**

Patients who underwent ACDF within one year postoperatively were enrolled in this cross-sectional study. Dysphagia symptoms were defined as an EAT-10 score ≥ 3. Swallowing function was evaluated using HRIM and videofluoroscopy. Swallowing tests were performed with two food textures, as defined by the International Dysphagia Diet Standardisation Initiative (IDDSI): level 0 (thin liquid) and level 4 (extremely thick liquid). HRIM parameters were compared between patients with and without dysphagia. Aspiration was defined as a Penetration–Aspiration Scale (PAS) score ≥ 6 on videofluoroscopic examination.

**Results:**

A total of 43 patients were included, comprising 29 with dysphagia symptoms and 14 without. During thin liquid swallowing (IDDSI level 0), patients with dysphagia symptoms had significantly lower hypopharyngeal mean peak pressure (hypoPeakP) and higher Swallow Risk Index (SRI) compared with those without dysphagia symptoms (median 55.79 vs. 104.85 mmHg and 18.60 vs. 4.30, respectively). During extremely thick liquid swallowing (IDDSI level 4), hypoPeakP remained significantly lower in the dysphagia symptoms group (median 86.96 vs. 161.19 mmHg), whereas SRI did not differ significantly between groups. No aspiration events were observed.

**Conclusions:**

Post-ACDF dysphagia symptoms were associated with reduced hypopharyngeal contractile pressure. These findings offer physiological insights into the association between dysphagia symptoms and swallowing biomechanics and may inform future research on swallowing assessment in this population.

**Trial registration:**

ClinicalTrials.gov Identifier: NCT04591665; IRB Approval: 202008024RINC.

**Supplementary Information:**

The online version contains supplementary material available at 10.1186/s13741-026-00691-4.

## Introduction

Anterior cervical discectomy and fusion (ACDF) is a commonly performed surgical procedure for the treatment of cervical disc herniation and spondylosis (Rhee and Ju 2016). Postoperative dysphagia symptoms are frequently reported and may be related to traction injury of the upper esophageal sphincter (UES) and surrounding swallowing muscles during surgical exposure of the cervical spine (Leonard and Belafsky 2011). Large database studies have reported dysphagia in approximately 14% of patients after ACDF (Cochran et al. 2026). Early identification of swallowing impairment may facilitate timely swallowing rehabilitation and reduce complications such as malnutrition and aspiration pneumonia (Altman et al. 2010).

Dysphagia symptoms evaluation typically incorporates both subjective and objective measures. The Eating Assessment Tool-10 (EAT-10) is a validated self-administered questionnaire widely used to screen for dysphagia symptoms (Belafsky et al. 2008). Objective assessments include videofluoroscopy and high-resolution impedance manometry (HRIM). Videofluoroscopy is widely considered the gold standard for identifying physiological impairments that may result in airway invasion and residue (Nijim et al. 2023). HRIM provides quantitative measurement of pharyngeal pressure and bolus transit through simultaneous pressure and impedance recordings (Lai et al. 2022; Omari et al. 2020, 2023). The combined use of videofluoroscopy and HRIM were used to provide information on swallowing physiology and the mechanisms underlying dysphagia.

Several studies have characterized postoperative dysphagia following ACDF using videofluoroscopy and patient-reported outcome measures (Haller et al. 2022). Videofluoroscopy has revealed altered pharyngeal function and swallowing biomechanics after ACDF (Muss et al. 2017; Miles et al. 2021; Jones-Rastelli et al. 2024; Kang et al. 2016; Leonard and Belafsky 2011). More recent investigations have compared videofluoroscopic findings with patient-reported measures such as the Eating Assessment Tool-10 (EAT-10), highlighting inconsistencies between physiologic impairment and symptom reporting (Jones-Rastelli et al. 2025). In addition, a recent scoping review emphasized the heterogeneity of assessment methods used to evaluate dysphagia after ACDF and suggested the need for more comprehensive physiologic characterization (Molfenter et al. 2023).

Despite growing recognition of postoperative dysphagia symptoms following ACDF, evidence regarding the manometric assessment of swallowing function using HRIM remains limited. Few studies have utilized HRIM to characterize swallowing biomechanics or have integrated patient-reported outcomes with videofluoroscopic findings in the same patients. Furthermore, the effects of varying liquid consistencies on swallowing biomechanics in this population are not yet fully understood.

To address these gaps, this study integrated HRIM with videofluoroscopy and patient-reported dysphagia symptoms to provide a more comprehensive characterization of swallowing biomechanics following ACDF. We aimed to investigate whether the severity of dysphagia symptoms, as measured by EAT-10 scores, is associated with objective alterations in swallowing biomechanics assessed by HRIM and videofluoroscopy.

## Materials and methods

This study was conducted in accordance with the ethical standards of the 1964 Declaration of Helsinki and was approved by the Institutional Review Board (IRB 202008024RINC) at our institution. Written informed consent was obtained from all participants.

### Participants

This was a prospective cross-sectional study conducted at a tertiary academic medical centre. Consecutive patients aged 20 to 80 years who had undergone ACDF were prospectively recruited from the neurosurgery outpatient clinic during the study period. The surgical technique was identical to that described in our previous study (Lai et al. 2022). At the time of evaluation, all participants were on oral intake and none required alternative feeding methods (e.g., nasogastric tube or gastrostomy) or had a tracheostomy. None of the participants had received prior instrumental swallowing assessment or formal swallowing rehabilitation before study enrollment. Postoperative swallowing assessments were performed between 1 month and 1 year after surgery. Patients were categorized into two groups based on EAT-10 scores: those with dysphagia symptoms (EAT-10 ≥ 3) and those without dysphagia symptoms (EAT-10 < 3). Patients were excluded if they met any of the following criteria : (1) underwent revision procedures; (2) were treated with surgery via both anterior and posterior approaches; (3) underwent ACDF for trauma, infection, tumor, or had any medical contraindications to local steroid infiltration; or (4) had coagulopathy.

### Fluid bolus preparations

The contrast medium was prepared by dissolving 5 g of sodium chloride in a commercially available electrolyte beverage (FIN, HeySong Corporation, Taiwan; total volume 580 mL) to standardize bolus conductivity for impedance measurements. After mixing, 180 mL of this solution was combined with Baritop LV powdered barium (300 g; 99.5% barium sulfate, Kaigen Pharma, Japan), resulting in a barium suspension of approximately 57.6% (w/w). Bolus consistency was defined according to the International Dysphagia Diet Standardization Initiative (IDDSI) framework (levels 0 to 7) (Rule et al. 2020); a thickening agent (Neo-High Toromeal III; Foodcare, Japan) was added as needed, and flow was confirmed using the IDDSI syringe test (10-second method)(Cheng et al. 2022). Two consistencies were tested: level 0 (thin liquid) and level 4 (extremely thick).

### Procedures

**Fig. 1:**
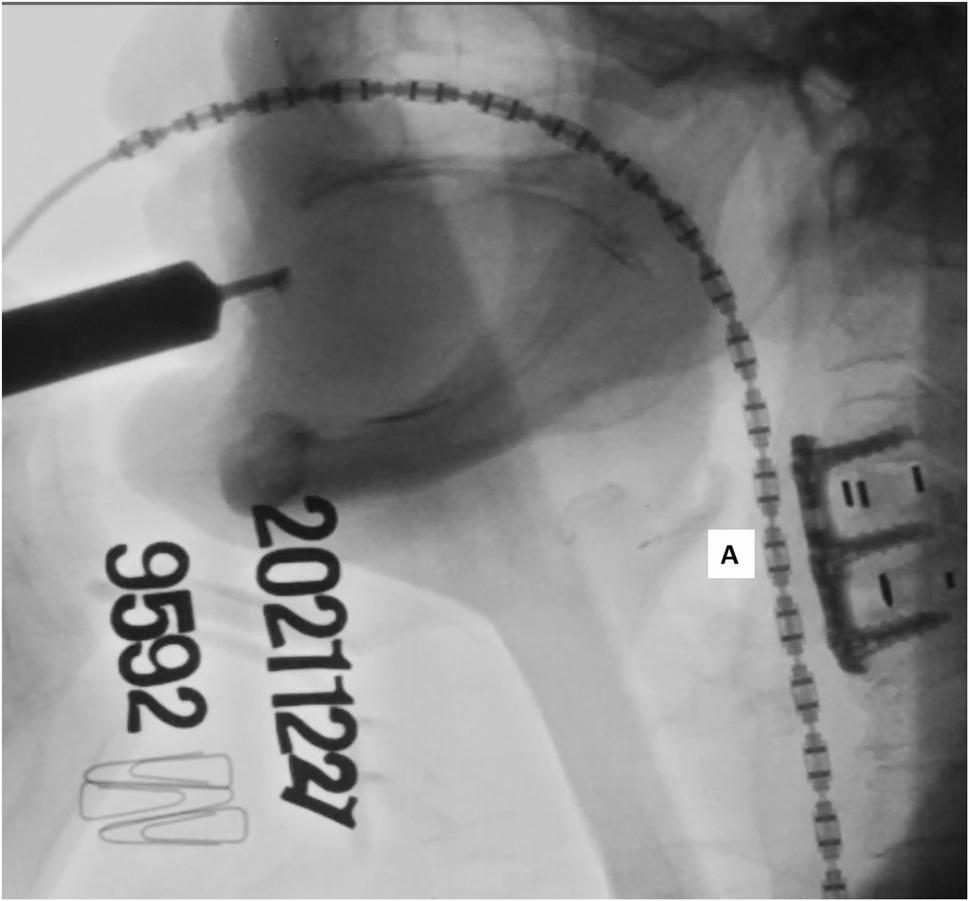
High-resolution impedance manometry (HRIM), with plate, and fluid bolus in the body is shown. Letter (**A**) indicates the point where the HRIM catheter traverses from the nasopharynx, oropharynx, and hypopharynx, through the upper esophageal sphincter into the esophagus

Patients who met the enrollment criteria completed the informed consent process and subsequently underwent simultaneous HRIM and videofluoroscopic examinations. After insertion of the HRIM catheter into the pharyngoesophageal segment (Fig. 1), each patient was positioned in a neutral sitting posture. Videofluoroscopy was performed using a Canon (formerly Toshiba) Ultimax-I DREX-UI80 system (Canon Medical Systems, Otawara, Japan) in continuous fluoroscopy mode. The fluoroscopic images were recorded and stored using a digital medical imaging system (MEDIRECO, TwinBeans Co., Ltd., Taiwan, ) and exported in MP4 format at 30 frames per second for frame-by-frame analysis. All videofluoroscopic recordings were independently reviewed by two experienced radiologists (Jo-Yu Chen and Yeun-Chung Chang). HRIM examinations were performed by an experienced physician (Chih-Jun Lai) trained in pharyngeal high-resolution impedance manometry. A solid-state catheter with a 10 Fr outer diameter, equipped with 36 circumferential pressure sensors spaced at 1-cm intervals and 12 impedance segments spaced at 2-cm intervals (MMS, Enschede, the Netherlands), was used. Before each recording, the catheter was calibrated to atmospheric pressure according to the manufacturer’s instructions. Patients fasted for at least 4 h prior to HRIM. Each patient swallowed a single 5-mL bolus for each texture (IDDSI level 0 and level 4).

### Outcome measurements

#### EAT-10

The EAT-10 questionnaire is a self report tool used to assess the severity of swallowing discomfort. It comprises 10 items, with participant responses recorded on a five-point Likert scale, ranging from 0 (no problem) to 4 (severe problem). The total score ranges from 0 to 40, with higher scores indicating more severe dysphagia symptoms. Patients were divided into two groups: those with dysphagia symptoms (positive; score ≥ 3) and those without dysphagia symptoms (negative; score < 3) (Belafsky et al. 2008).

#### HRIM data analysis

The manometry data were analyzed performed utilizing the methodology described in our prior study (Lai et al. 2022). HRIM metrics (Table 1) were selected for the comparative analysis of swallows by using MATLAB (MathWorks in Natick, MA, USA) (Lai et al. 2022; Schar et al. 2020, 2022; Cock and Omari 2017), including: (1) UES 0.25s integrated relaxation pressure (mmHg), (2) UES basal pressure (mmHg) ; (3) UES post-deglutitive peak pressure (UES mmHg); (4) UES open time (s); (5) UES maximum admittance (UES Adm, mS); (6) Mesopharyngeal contractile (mmHg.s.cm); (7) hypopharyngeal mean peak pressure (hypoPeakP, mmHg); (8) hypopharyngeal intrabolus pressure at 1 cm above the UES (mmHg); (9) hypopharyngeal distention contraction latency (ms); (10) hypopharyngeal bolus presence time (s); (11) velopharyngeal contractile (mmHg.s.cm); (12) swallow risk index (SRI). The SRI was determined using the following formula (Lai et al. 2022; Schar et al. 2020): SRI = (IBP × BPT)/ ((DCL + 1) × hypoPeakP) × 100.

**Table 1:** Clinical meanings of HRIM parameters

Class	Metric	Acronym	Normative range	Unit	Interpretation
Pharyngeal metrics	Velopharyngeal contractile integral	VCI	17 to 179	mmHg.cm.s	A decrease in values reflects impaired function of the velo-, or mesopharyngeal muscles
	Mesopharyngeal contractile integral	MCI	40 to 233		
	Hypopharyngeal mean peak pressure	hypoPeakP	69 to 280	mmHg	A reduced value reflects diminished contractility of the hypopharynx
	Hypopharyngeal distention contraction latency	DCL	317 to 598	ms	The duration between the maximum opening of the hypopharyngeal muscles and their peak contraction during bolus passage through the hypopharyngeal region
	Intra-bolus pressure	IBP	-1 to 22	mmHg	An increased value reflects greater UES resistance
UES metrics	UES maximum admittance	UES Max Adm	4.4 to 9.1	mS	Lower values suggest a restricted UES opening extent
	UES integrated relaxation pressure	UES IRP	-4 to 15	mmHg	An increase in value reflects UES impaired relaxation and constriction.
	UES open time	UES open time	0.4 to 0.7	**s**	Decreased values imply a limited duration of UES relaxation
	UES postdeglutitive peak pressure	PeakP	69 to 280	mmHg	Decreased values indicate decreased UES contractility
	UES basal pressure	UES BP	76 to 268	mmHg	A reduced value indicates diminished pre-deglutitive tone
Bolus presence time	Bolus presence time	BPT	0.4 to 0.9	s	A lower value means the bolus presence in the pharynx for less time prior to swallowing initiation
Global measure of swallow function	Swallow Risk Index	SRI	Aspiration risk > 15		An increased value reflects a more extensive impairment in swallowing function

*UES *upper esophageal sphincter, *mS *S was calculated to be 1/Ω, *mS *S × 1,000, *ms *millisecond, *s *second

a:5mL thin (IDDSI 0) fluids (reference: Cock and Omari 2017)

#### The Penetration Aspiration Scale (PAS) and the Bolus Residue Scale

The Penetration–Aspiration Scale (PAS) is an 8-point ordinal scale used to quantify the depth of airway invasion and the patient’s response to swallowed material (including fluid or solid) during instrumental swallowing assessment (Rosenbek et al. 1996). PAS scores of 3–5 indicate penetration, whereas scores of 6–8 indicate aspiration events. In this study, aspiration events were defined as a PAS score ≥ 6. Additionally, pharyngeal residue was graded using the Bolus Residue Scale (BRS), which ranges from 1 (no residue) to 6 (residue present in the vallecula, posterior pharyngeal wall, and piriform sinuses (Rommel et al. 2015). On this scale, a score of 1 indicates no residue, and scores greater than 1 indicate the presence of pharyngeal residue (Rommel et al. 2015).

### Statistical analysis

Categorical variables were presented as numbers (percentages) and analyzed using Pearson’s chi-squared test or Fisher’s exact test. Continuous variables were first tested for normality using the Shapiro–Wilk test. Normally distributed variables are reported as mean (standard deviation), whereas non-normally distributed variables are reported as median (Q1–Q3). Patients were divided into two groups for comparison: those with dysphagia symptoms (EAT-10 ≥ 3) and those without dysphagia symptoms (EAT-10 < 3). Group comparisons were conducted using the Student’s t-test or the Mann–Whitney U test. Statistical significance was defined as *P* < 0.05. The relationship between HRIM parameters and dysphagia was examined using linear regression analyses. The 12 HRIM parameters included hypopharyngeal mean peak pressure, velopharyngeal contractile, mesopharyngeal contractile, UES basal pressure, UES 0.25 relaxing pressure, UES peak pressure, UES open time, UES admittance, bolus presence time, hypopharyngeal intrabolus pressure at 1 cm above the UES, hypopharyngeal distention contraction latency, and the SRI. For multivariate analysis, we constructed 12 multiple linear regression models adjusting for patients with and without dysphagia symptoms, age, sex, BMI, Charlson Comorbidity Index, hypertension, diabetes, smoking, drinking, numbers of cervical level fused, and the highest of cervical levels for each HRIM parameter. All analyses were performed using R Statistical Software (v4.0.3; R Core Team 2020). Additionally, inter- and intra-rater reliability of PAS and BRS scoring from videofluoroscopy were assessed using weighted kappa statistics. Intra-rater reliability was evaluated with the rater blinded to the original ratings. For HRIM, intra-rater reliability of hypopharyngeal mean peak pressure (hypoPeakP), the primary parameter identified in our previous study (Lai et al. 2022), was evaluated by the same trained physician using the intraclass correlation coefficient (ICC).

## Results

**Fig. 2:**
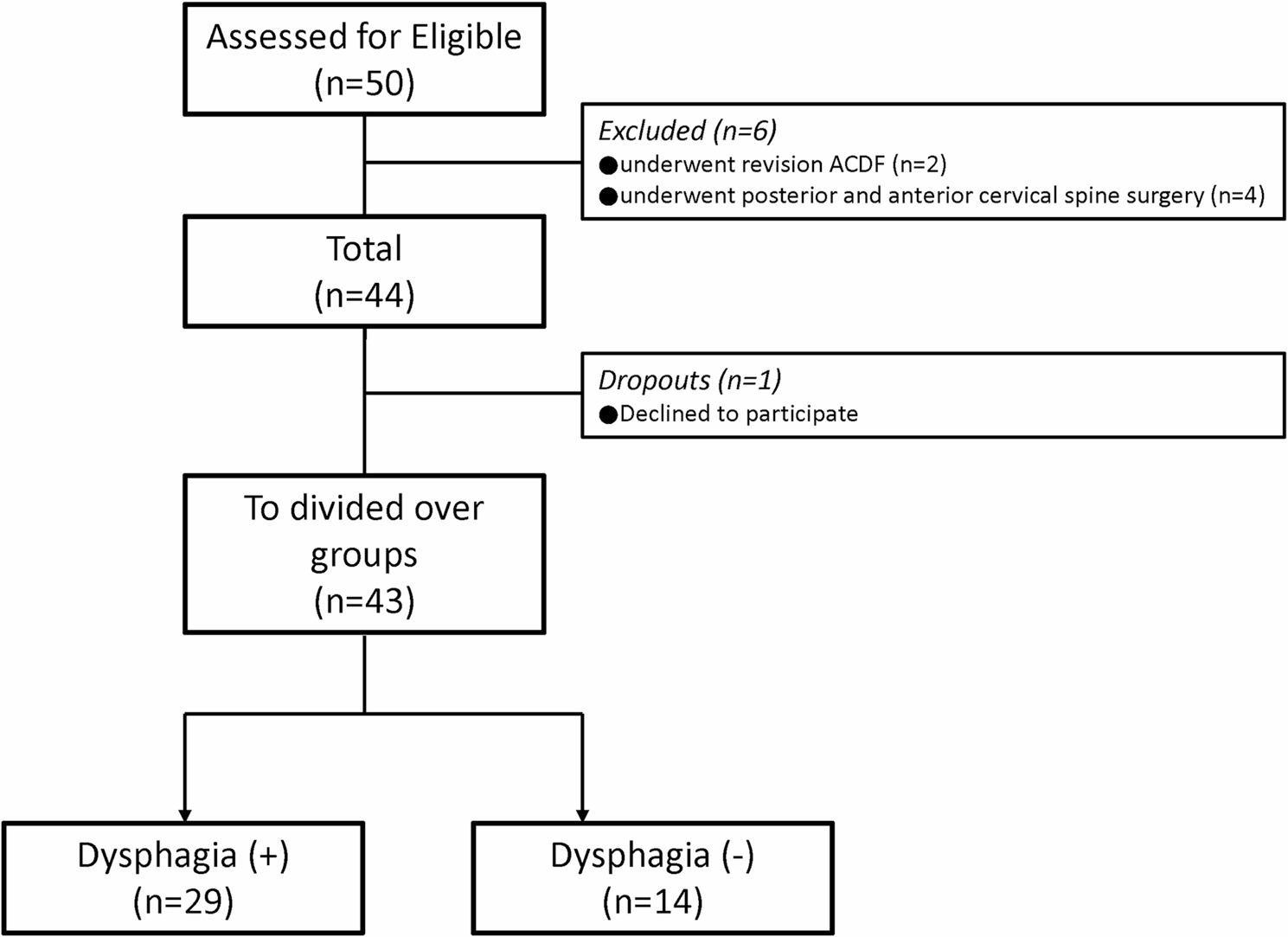
Flow diagram illustrating the process of patient recruitment and analysis

Fifty patients were initially eligible; six were excluded for not meeting the inclusion criteria, and one declined participation, leaving 43 patients for analysis (Fig. 2). Of these, 29 (67.4%) had dysphagia symptoms (EAT-10 ≥ 3) and 14 (32.6%) did not. The mean age was 57.49 ± 13.91 years (range: 26–79), with 20 males and 23 females. The median [Q1, Q3] EAT-10 score was 5.00 [2.00, 10.75], with a range of 0–38. Three patients had scores between 30 and 40. Demographic characteristics are presented in Table 2. The distribution of postoperative follow-up intervals did not differ significantly between patients with and without dysphagia symptoms (Supplementary Table 1).

**Table 2:** Demographic data between patients with/without dysphagia

	Dysphagia symptoms (-)	Dysphagia symptoms (+)	
	*n* = 14	*n* = 29	*P*-value
Age, No. (%)			0.93
<65 years	8 (57.14%)	17 (58.62%)	
≥65 years	6 (42.86%)	12 (41.38%)	
Sex, No. (%)			0.75
Male	7 (50.00%)	13 (44.83%)	
Female	7 (50.00%)	16 (55.17%)	
Body Mass Index, No. (%)			0.93
<25	8 (57.14%)	17 (58.62%)	
≥25	6 (42.86%)	12 (41.38%)	
Charlson Comorbidity Index, No. (%)			0.93
<3	8 (57.14%)	17 (58.62%)	
≥3	6 (42.86%)	12 (41.38%)	
Hypertension, No. (%)			0.59
No	6 (42.86%)	15 (51.72%)	
Yes	8 (57.14%)	14 (48.28%)	
Diabetes, No. (%)			0.02
No	7 (50.00%)	25 (86.21%)	
Yes	7 (50.00%)	4 (13.79%)	
Smoking, No. (%)			1.00
No	12 (85.71%)	26 (89.66%)	
Yes	2 (14.29%)	3 (10.34%)	
Alcohol consumption, No. (%)			1.00
No	13 (92.86%)	28 (96.55%)	
Yes	1 (7.14%)	1 (3.45%)	
Numbers of cervical level fused, No. (%)			0.89
<2	5 (35.71%)	11 (37.93%)	
≥2	9 (64.29%)	18 (62.07%)	
The highest of cervical levels, No. (%)			0.84
C3/4	5 (35.71%)	11 (37.93%)	
C4/5	4 (28.57%)	6 (20.69%)	
C5/6	5 (35.71%)	12 (41.38%)	

At IDDSI level 0 (thin liquid), patients with dysphagia symptoms had significantly lower hypopharyngeal mean peak pressure measured by HRIM compared to those without dysphagia symptoms, indicating reduced hypopharyngeal muscle strength (median [Q1, Q3]: 55.79 [43.02, 67.50] vs. 109.24 [72.73, 162.21] mmHg, *p* < 0.01). In the multivariable linear regression model, dysphagia symptoms remained independently associated with lower hypopharyngeal mean peak pressure (β = −78.37, 95% CI [− 127.01, − 29.73], *p* < 0.01) (Table 3). Additionally, the SRI was significantly higher in patients with dysphagia symptoms than in those without dysphagia symptoms (median [Q1, Q3]: 18.60 [7.53, 42.31] vs. 4.30 [− 2.32, 7.05], *p* < 0.01). In the multivariable analysis, dysphagia symptoms remained independently associated with higher SRI (β = 20.97, 95% CI [5.32, 36.63], *p* = 0.01).

**Table 3:** HRIM related parameters using IDDSI 0 of patients with and without dysphagia

Outcomes, Median [Q1, Q3]	Dysphagia symptoms (-)	Dysphagia symptoms (+)	Univariate analysis		Multivariate analysis	
	*n* = 14	*n* = 29	β (95% CI)	*P*-value	β (95% CI)	*P*-value
Hypopharyngeal mean peak pressure, mmHg	104.85 [71.17, 157.23]	55.79 [43.02, 67.50]	-75.68 (-119.48, -31.88)	< 0.01	-78.37 (-127.01, -29.73)	< 0.01
Velopharyngeal contractile, mmHg.s.cm	71.04 [38.51, 161.47]	70.25 [35.67, 162.67]	13.88 (-46.26, 74.03)	0.64	44.65 (-15.35, 104.646)	0.14
Mesopharyngeal contractile, mmHg.s.cm	291.24 [149.17, 414.04]	261.85 [145.17, 370.31]	10.73 (-108.45, 129.91)	0.86	39.42 (-93.51, 172.35)	0.55
UES basal pressure, mmHg	40.26 [35.34, 75.12]	45.80 [27.54, 105.53]	21.63 (-13.45, 56.72)	0.22	13.69 (-27.41, 54.78)	0.50
UES 0.25 relaxing pressure, mmHg	14.13 [5.89, 39.54]	25.82 [17.51, 38.92]	9.19 (-2.19, 20.57)	0.11	9.64 (-3.36, 22.63)	0.14
UES peak pressure, mmHg	161.00 [94.95, 187.34]	157.02 [117.45, 218.06]	38.75 (-49.13, 126.64)	0.38	31.19 (-67.37, 129.75)	0.52
UES open time, s	0.89 [0.77,1.06]	0.87 [0.80, 1.03]	0.02 (-0.15, 0.18)	0.85	0.01 (-0.16, 0.18)	0.91
UES admittance, mS	2.95 [2.65, 3.73]	3.44 [2.83, 3.63]	0.18 (-0.27, 0.62)	0.43	0.03 (-0.45, 0.52)	0.89
Bolus presence time, s	0.74 [0.54, 1.03]	0.78 [0.64,0.93]	0.05 (-0.14, 0.23)	0.62	0.08 (-0.14, 0.29)	0.48
Hypopharyngeal intrabolus pressure at 1 cm above the UES, mmHg	8.38 [-4.00, 15.73]	15.77 [8.47, 35.01]	25.57 (-0.60, 51.73)	0.06	23.09 (-7.91, 54.09)	0.14
Hypopharyngeal distention contraction latency, ms	289.82 [251.51, 395.42]	323.14 [254.64, 426.64]	28.80 (-47.96, 105.57)	0.45	19.12 (-68.75, 107.99)	0.66
Swallowing risk index	4.30 [-2.32, 7.05]	18.60 [7.53, 42.31]	20.79 (7.82, 33.76)	< 0.01	20.97 (5.32, 36.63)	0.01

*UES *upper esophageal sphincter, *HRIM *high resolution impedance manometry

At IDDSI level 4 (extremely thick liquid), patients with dysphagia symptoms had lower hypopharyngeal mean peak pressure measured by HRIM compared to those without dysphagia symptoms (median [Q1, Q3]: 86.96 [50.30, 119.58] vs. 161.19 [106.52, 205.73] mmHg, *p* < 0.01). In the multivariable analysis, dysphagia symptoms remained independently associated with lower hypopharyngeal mean peak pressure (β = −64.09, 95% CI [− 112.08, − 16.12], *p* = 0.01) (Table 4). Additionally, the SRI was significantly higher in patients with dysphagia symptoms than in those without dysphagia symptoms (median [Q1, Q3]: 12.18 [4.63, 25.21] vs. 4.85 [0.35, 8.60], *p* = 0.03). However, SRI was not significantly associated with dysphagia symptoms after adjustment (Table 4).

**Table 4:** HRIM related parameters using IDDSI 4 between patients with and without dysphagia

Outcomes, Median [Q1, Q3]	Dysphagia symptoms (-)	Dysphagia symptoms (+)	Univariate analysis		Multivariate analysis	
	*N* = 14	*N* = 29	β (95% CI)	*P*-value	β (95% CI)	*P*-value
Hypopharyngeal mean peak pressure, mmHg	161.19 [106.52, 205.73]	86.96 [50.30, 119.58]	-61.07 (-103.81, -18.33)	< 0.01	-64.09 (-112.08, -16.12)	0.01
Velopharyngeal contractile, mmHg.s.cm	55.85 [13.95, 149.72]	107.74 [54.71, 171.01]	59.42 (-6.31, 125.14)	0.08	76.34 (-1.02, 153.69)	0.05
Mesopharyngeal contractile, mmHg.s.cm	357.37 [145.98, 468.54]	221.51 [163.05, 313.37]	-3.13 (-155.26, 148.99)	0.97	5.23 (-160.08, 170.55)	0.95
UES basal pressure, mmHg	36.56 [18.41, 66.23]	48.00 [21.39, 66.49]	-0.57 (-29.17, 28.03)	0.97	6.13 (-25.88, 38.15)	0.70
UES 0.25 relaxing pressure, mmHg	13.09 [4.20, 28.21]	30.59 [18.57, 42.84]	11.16 (1.04, 21.28)	0.03	10.27 (-1.20, 21.74)	0.08
UES peak pressure, mmHg	172.06 [118.54, 307.48]	184.48 [107.48, 293.22]	23.76 (-73.37, 120.89)	0.62	19.52 (-81.95, 121.00)	0.70
UES open time, s	0.94 [0.77, 1.04]	0.86 [0.77, 1.13]	0.03 (-0.14, 0.20)	0.74	0.03 (-0.15, 0.21)	0.74
UES admittance, mS	3.65 [3.01, 4.24]	3.99 [3.28, 4.51]	0.27 (-0.40, 0.95)	0.42	0.20 (-0.59, 0.98)	0.62
Bolus presence time, s	0.73 [0.52, 0.77]	0.74 [0.54, 0.91]	0.08 (-0.12, 0.28)	0.43	0.08 (-0.14, 0.30)	0.47
Hypopharyngeal intrabolus pressure at 1 cm above the UES, mmHg	13.05 [2.48, 22.95]	17.21 [8.42, 33.12]	10.26 (-26.38, 46.91)	0.58	3.77 (-38.48, 46.03)	0.86
Hypopharyngeal distention contraction latency, ms	372.00 [300.00, 444.22]	333.48 [285.65, 405.87]	-17.89 (-97.87, 62.10)	0.65	-1.28 (-87.65, 85.09)	0.98
Swallowing risk index	6.63 [1.51, 9.73]	12.18 [4.63, 25.21]	10.18 (-3.41, 23.78)	0.14	11.12 (-5.33, 27.57)	0.18

*UES *upper esophageal sphincter, *HRIM *high resolution impedance manometry

No aspiration events were observed during videofluoroscopic evaluation (PAS ≥ 6) in either patients with or without dysphagia symptoms across IDDSI levels 0 and 4. At IDDSI level 0, PAS scores did not differ significantly between patients with and without dysphagia symptoms (median [Q1, Q3]: 1 [1, 1] vs. 1 [1, 1], *p* = 0.43). Similarly, at IDDSI level 4, PAS scores were comparable between the two groups (median [Q1, Q3]: 1 [1, 1] vs. 1 [1, 1], *p* = 0.78). PAS scores ranged from 1 to 5 across all groups at both IDDSI levels. Median BRS scores were 1.00 in both groups, and no significant differences were observed between patients with and without dysphagia symptoms at either IDDSI level 0 or 4. Residue (BRS > 1) was observed in 3/29 and 2/29 patients with dysphagia symptoms at IDDSI levels 0 and 4, respectively, but in none of the patients without dysphagia symptoms (Supplementary Table 2).

Reliability analysis was conducted for the videofluoroscopic parameters (PAS and BRS) and the HRIM parameter, hypopharyngeal mean peak pressure, with reliability coefficients ranging from 0.70 to 0.99 across IDDSI levels (Supplementary Table 3).

## Discussion

This study demonstrated that patients with dysphagia symptoms following ACDF exhibited significantly reduced hypopharyngeal muscle strength at both IDDSI level 0 (thin liquid) and IDDSI level 4 (extremely thick liquid). No aspiration events were observed during videofluoroscopic evaluation.

In our study, patients with persistent dysphagia symptoms were evaluated between 1 month and 1 year postoperatively. Our previous research demonstrated partial recovery of hypopharyngeal contractility within the first postoperative week (Lai et al. 2022), suggesting that recovery varies among patients, with some improving early and others experiencing persistent symptoms. Early reduced hypopharyngeal contractility following ACDF is thought to be primarily driven by surgical trauma–related factors, including soft tissue edema, local inflammation, and mechanical traction injury to the upper esophageal sphincter (UES) during surgical retraction. These transient changes are typically observed in the immediate postoperative period and often improve within the first few days (Leonard and Belafsky 2011; Kang et al. 2016). Persistent dysphagia symptoms associated with reduced hypopharyngeal contractility observed in our study may reflect longer-term alterations in swallowing biomechanics, including impaired pharyngeal constriction (Miles et al. 2021), or incomplete neural recovery involving the superior laryngeal nerve, pharyngeal plexus, or recurrent laryngeal nerve (Winslow et al. 2001). However, these proposed mechanisms remain speculative and require further investigation.

Notably, the reduced hypopharyngeal mean peak pressure observed in our study may indicate decreased contractile strength of the pharyngeal constrictor muscles (Cock and Omari 2017). Previous studies have shown that pharyngeal pressure is closely associated with fluoroscopic measures of pharyngeal movement and bolus clearance (Pauloski et al. 2009), suggesting that reduced pressure may contribute to impaired pharyngeal contraction and swallowing efficiency. Physiologic indices such as the SRI, derived from multiple hypopharyngeal pressure–flow metrics, may therefore reflect alterations in swallowing biomechanics and bolus clearance efficiency rather than provide direct evidence of airway invasion. This interpretation is consistent with our findings, in which only a small proportion of patients with dysphagia symptoms exhibited pharyngeal residue, and no airway invasion was observed. Although pharyngeal residue is considered a potential risk factor for aspiration, the limited degree of residue observed in our patients may not have been sufficient to compromise airway safety (Cock and Omari 2017).

Hypopharyngeal muscle strength and coordination play a critical role in effective bolus propulsion during swallowing (Schindler and Kelly 2002). Quantitative assessment of hypopharyngeal contractility may therefore help identify patients at risk of dysphagia. However, evidence remains limited (Omari et al. 2020), and no widely accepted cutoff exists for patients undergoing ACDF, particularly given variability across bolus consistencies and testing conditions. In our study, patients with dysphagia symptoms exhibited lower hypopharyngeal mean peak pressure during both thin (median 55.79 mmHg) and extremely thick liquid swallowing (median 86.96 mmHg). These findings suggest that a relative reduction in pharyngeal contractility is a hallmark of postoperative functional impairment. Although the data demonstrates a consistent pattern of contractile insufficiency in symptomatic individuals, establishing a definitive absolute pressure threshold as a diagnostic cutoff will require further empirical validation. Therefore, hypopharyngeal pressure should be interpreted in conjunction with clinical symptoms and complementary videofluoroscopic findings. Further studies are needed to establish clinically meaningful thresholds for guiding intervention.

Previous studies have explored rehabilitative approaches targeting pharyngeal muscle function, including effortful swallow and related exercises (Ko et al. 2022). In the present study, we focused on the physiological characterization of swallowing function and did not evaluate therapeutic interventions. Therefore, further research is warranted to determine whether such approaches may influence hypopharyngeal contractility in this population. A major strength of this study is the provision of objective physiological data demonstrating an association between hypopharyngeal contractility and patient-reported dysphagia symptoms after ACDF. The EAT-10, a validated screening tool, can be easily administered by healthcare providers or nurses. An EAT-10 score ≥ 3 reflects greater dysphagia symptom burden and was associated with alterations in swallowing biomechanics in this study, providing clinically relevant insights into the relationship between symptoms and physiology.

This study has several limitations. First, cross-sectional design limits the ability to determine causal relationships. Second, the small sample size may reduce the generalizability of the findings. Future studies with larger post-ACDF cohorts are needed to enable comprehensive swallowing assessments and clarify causal effects. Third, a single experienced physician performed HRIM analysis. Although HRIM parameters are objectively derived using standardized analytical methods, the absence of inter-rater reliability assessment remains a limitation. Fourth, given the small sample size in this pilot study, correction for multiple comparisons was not applied, and the possibility of overfitting cannot be excluded. Therefore, the findings should be interpreted with caution. Future studies with larger sample sizes are warranted to validate these results. Fifth, normative HRIM reference values for IDDSI level 4 remain incompletely established and require further investigation. Finally, all patients included in this study had normal PAS scores based on videofluoroscopy, which further limits the generalizability of the findings. Future studies are warranted to evaluate HRIM findings in patients with airway compromise or elevated PAS scores to better understand the broader applicability of these results.

## Conclusions

Post-ACDF dysphagia symptoms were associated with reduced hypopharyngeal muscle function. Our findings provide physiological insights into the associations between dysphagia symptoms (EAT-10 ≥ 3) and swallowing biomechanics. These results may inform future research on the evaluation of dysphagia symptoms in patients undergoing ACDF.

## Supplementary Information

Supplementary Material 1.

## Data Availability

The datasets used and analyzed during the current study are available from the corresponding author upon reasonable request.

## References

[CR1] Altman KW, Yu GP, Schaefer SD. Consequence of Dysphagia in the Hospitalized Patient: Impact on Prognosis and Hospital Resources. Arch Otolaryngol Head Neck Surg. 2010;136(8):784–9. 20713754 10.1001/archoto.2010.129

[CR2] Belafsky PC, Mouadeb DA, Rees J, et al. Validity and reliability of the Eating Assessment Tool (EAT-10). Ann Otol Rhinol Laryngol. 2008;117(12):919–24. 19140539 10.1177/000348940811701210

[CR3] Cheng CH, Chen HC, Chen JY, et al. The standardizing texture of thickened barium stimuli in the videofluoroscopic swallowing study at a medical center in Taiwan. J Formos Med Assoc. 2022;121(2):563–5. 34348866 10.1016/j.jfma.2021.07.020

[CR4] Cochran J, Deng N, Tabbaa A, Razi A, Abu-Ghanem S. Dysphagia Following Anterior Cervical Discectomy and Fusion: A PearlDiver Analysis of Incidence, Risk Factors, and Interventions. Dysphagia. 2026;41(1):171–8. 40775465 10.1007/s00455-025-10867-7

[CR5] Cock C, Omari T. Diagnosis of Swallowing Disorders: How We Interpret Pharyngeal Manometry. Curr Gastroenterol Rep. 2017;19(3):11. 28289859 10.1007/s11894-017-0552-2 PMC5348549

[CR6] Haller L, Kharidia KM, Bertelsen C, Wang J, O’Dell K. Post-Operative Dysphagia in Anterior Cervical Discectomy and Fusion. Ann Otol Rhinol Laryngol. 2022;131:289–94. 34075815 10.1177/00034894211015582

[CR8] Jones-Rastelli RB, Amin MR, Balou M, Herzberg G, Molfenter S. Alterations in Swallowing Six Weeks After Primary Anterior Cervical Discectomy and Fusion (ACDF). Dysphagia. 2024;39(4):684–96. 38157009 10.1007/s00455-023-10649-z PMC12243682

[CR7] Jones-Rastelli RB, Amin MR, Anandhakrishnan M, et al. Comparing Videofluoroscopic and Patient Reported Outcome Measures of Swallowing After ACDF Surgery. Laryngoscope. 2025;135:3804–14. 40719035 10.1002/lary.32352 PMC13526359

[CR9] Kang SH, Kim DK, Seo KM, et al. Swallowing Function Defined by Videofluoroscopic Swallowing Studies after Anterior Cervical Discectomy and Fusion: a Prospective Study. J Korean Med Sci. 2016;31(12):2020–25. 27822944 10.3346/jkms.2016.31.12.2020 PMC5102869

[CR10] Ko JH, Han KS, Yoon SJ. Efficacy of Laryngeal Rehabilitation Therapy on Dysphagia after Anterior Cervical Surgery: Prospective, Randomized Control Trial. J Clin Med. 2022;11(9):2470. 35566596 10.3390/jcm11092470 PMC9102732

[CR11] Lai CJ, Cheng YJ, Lai DM, et al. Applying High-Resolution Impedance Manometry for Detecting Swallowing Change in Anterior Cervical Spine Surgery Patients. Front Surg. 2022;9:851126. 35372473 10.3389/fsurg.2022.851126 PMC8965755

[CR12] Leonard R, Belafsky P. Dysphagia following cervical spine surgery with anterior instrumentation: evidence from fluoroscopic swallow studies. Spine. 2011;36:2217–23. 21325988 10.1097/BRS.0b013e318205a1a7

[CR13] Miles A, Jamieson G, Shasha L, Davis K. Characterizing dysphagia after spinal surgery. J Spinal Cord Med. 2021;44(5):733–41. 31549950 10.1080/10790268.2019.1665613 PMC8477967

[CR14] Molfenter SM, Amin MR, Balou M, Herzberg EG, Frempong-Boadu A. A scoping review of the methods used to capture dysphagia after anterior cervical discectomy and fusion: the need for a paradigm shift. Eur Spine J. 2023;32(3):969–76. 36625955 10.1007/s00586-022-07515-1 PMC10805127

[CR15] Muss L, Wilmskoetter J, Richter K, et al. Changes in Swallowing After Anterior Cervical Discectomy and Fusion With Instrumentation: A Presurgical Versus Postsurgical Videofluoroscopic Comparison. J Speech Lang Hear Res. 2017;60(4):785–93. 28319639 10.1044/2016_JSLHR-S-16-0091

[CR16] Nijim W, Cowart JH, Banerjee C, Postma G, Paré M. Evaluation of outcome measures for post-operative dysphagia after anterior cervical discectomy and fusion. Eur Arch Otorhinolaryngol. 2023;280(11):4793–801. 37592082 10.1007/s00405-023-08167-7

[CR18] Omari TI, Ciucci M, Gozdzikowska K, et al. High-Resolution Pharyngeal Manometry and Impedance: Protocols and Metrics-Recommendations of a High-Resolution Pharyngeal Manometry International Working Group. Dysphagia. 2020;35(2):281–95. 31168756 10.1007/s00455-019-10023-y

[CR17] Omari T, Cock C, Wu P, et al. Using high resolution manometry impedance to diagnose upper esophageal sphincter and pharyngeal motor disorders. Neurogastroenterol Motil. 2023;35(1):e14461. 36121685 10.1111/nmo.14461

[CR19] Pauloski BR, Rademaker AW, Lazarus C, et al. Relationship between manometric and videofluoroscopic measures of swallow function in healthy adults and patients treated for head and neck cancer with various modalities. Dysphagia. 2009;24(2):196–203. 18956228 10.1007/s00455-008-9192-x PMC2892906

[CR20] Rhee JM, Ju KL. Anterior Cervical Discectomy and Fusion. JBJS Essent Surg Tech. 2016;6(4):e37. 30233930 10.2106/JBJS.ST.15.00056 PMC6132613

[CR21] Rommel N, Borgers C, Beckevoort DV, et al. Bolus Residue Scale: An Easy-to-Use and Reliable Videofluoroscopic Analysis Tool to Score Bolus Residue in Patients with Dysphagia. Int J Otolaryngol. 2015;2015:780197. 26640491 10.1155/2015/780197 PMC4660024

[CR22] Rosenbek JC, Robbins JA, Roecker EB, Coyle JL, Wood JL. A penetration-aspiration scale. Dysphagia. 1996;11:93–8. 8721066 10.1007/BF00417897

[CR23] Rule DW, Kelchner L, Mulkern A, et al. Implementation Strategies for the International Dysphagia Diet Standardisation Initiative (IDDSI), Part I: Quantitative Analysis of IDDSI Performance Among Varied Participants. Am J Speech Lang Pathol. 2020;29(3):1514–28. 32510986 10.1044/2020_AJSLP-19-00012

[CR24] Schar SM, Omari TI, Fraser RJ, Bersten AD, Bihari S. Disordered swallowing associated with prolonged oral endotracheal intubation in critical illness. Intensive Care Med. 2020;46(1):140–2. 31713057 10.1007/s00134-019-05844-2

[CR25] Schar SM, Omari TI, Woods CM, et al. Pharyngeal tongue base augmentation for dysphagia therapy: A prospective case series in patients post head and neck cancer treatment. Head Neck. 2022;44:1871–84. 35665556 10.1002/hed.27104

[CR26] Schindler JS, Kelly JH. Swallowing disorders in the elderly. Laryngoscope. 2002;112(4):589–602. 12150508 10.1097/00005537-200204000-00001

[CR27] Winslow CP, Winslow TJ, Wax MK. Dysphonia and Dysphagia Following the Anterior Approach to the Cervical Spine. Arch Otolaryngol Head Neck Surg. 2001;127(1):51–5. 11177014 10.1001/archotol.127.1.51

