# Supplementary material for: Reduced hypopharyngeal muscle strength in patients with dysphagia symptoms after anterior cervical discectomy and fusion
Source: Perioper Med (Lond). 2026 May 7;15:59. doi: 10.1186/s13741-026-00691-4 (PMC13317437; doi:10.1186/s13741-026-00691-4)
Supplement: Supplementary file 1 — Supplementary Material 1. [file 13741_2026_691_MOESM1_ESM.docx]

**Supplementary Table 1 Follow up time**

|  | **Dysphagia symptoms (-)** | **Dysphagia symptoms (+)** | **P value** |
| --- | --- | --- | --- |
| Variables, n (%) | N=14 | N=29 |  |
| Time duration |  |  | 1.0 |
| 1-3 months | 10 (71.43%) | 20 (68.96%) |  |
| ≥3 month to 1 year | 4 (28.57%) | 9 (31.04%) |  |

Data are presented as n (%).

**Supplementary Table 2 Bolus Residue Scale (BRS) and residue (BRS>1) between patients with or without dysphagia symptoms**

|  |  | Dysphagia symptoms (-) | Dysphagia symptoms (+) | P value |
| --- | --- | --- | --- | --- |
|  |  | N=14 | N=29 |  |
| IDDSI 0 | BRS | 1.00 [1.00,1.00] | 1.00 [1.00,1.00] | 0.22 |
|  | Residue (BRS>1) | 0 (0.00%) | 3 (10.34%) | 0.54 |
| IDDSI 4 | BRS | 1.00 [1.00,1.00] | 1.00 [1.00,1.00] | 0.32 |
|  | Residue (BRS>1) | 0 (0.00%) | 2 (6.89%) | 1.00 |

Data are presented as median [Q1, Q3] or n (%). BRS: Bolus Residue Scale; IDDSI: International Dysphagia Diet Standardization Initiative

**Supplementary Table 3: Intra and inter reliability of hypopharyngeal mean peak pressure from HRIM and PAS and BRS scores from videofluoroscopy**

| Instrument | Reliability | Parameter | IDDSI | Reliability coefficient (95% CI) |
| --- | --- | --- | --- | --- |
|  |  |  |  | ICC (95%CI) |
| HRIM | Intra-rater reliability | Hypopharyngeal mean peak pressure | IDDSI 0 | 0.99 [0.97,1.00] |
|  |  |  | IDDSI 4 | 0.96[0.98,0.99] |
|  |  |  |  | weighted kappa (95%CI) |
| Videofluoroscopy | Intra-rater reliability | PAS | IDDSI 0 | 0.96 [0.94, 0.98] |
|  |  |  | IDDSI 4 | 0.97[0.93,1.00] |
|  | Inter-rater reliability | PAS | IDDSI 0 | 0.92 [0.82, 1.00] |
|  |  |  | IDDSI 4 | 0.94 [0.83, 0.94] |
|  | Intra-rater reliability | BRS | IDDSI 0 | 0.79[0.39,1.00] |
|  |  |  | IDDSI 4 | 0.72[0.56,0.87] |
|  | Inter-rater reliability | BRS | IDDSI 0 | 0.70[0.46,0.95] |
|  |  |  | IDDSI 4 | 0.79[0.39,1.00] |

HRIM: high resolution impedance manometry; PAS: penetration aspiration scale; BRS: Bolus Residue Scale; IDDSI: International Dysphagia Diet Standardization Initiative; ICC: Intraclass Correlation Coefficient
